# Identification and estimation of the intake of fermented foods and their contribution to energy and nutrients among Japanese adults

**DOI:** 10.1017/S1368980024000405

**Published:** 2024-02-16

**Authors:** Hitomi Fujihashi, Satoshi Sasaki

**Affiliations:** 1 Department of Social and Preventive Epidemiology, Graduate School of Medicine, The University of Tokyo, 7-3-1 Hongo, Bunkyo-ku, Tokyo 113-0033, Japan; 2 Department of Social and Preventive Epidemiology, School of Public Health, The University of Tokyo, 7-3-1 Hongo, Bunkyo-ku, Tokyo 113-0033, Japan

**Keywords:** Fermentation, Fermenting, Consumption, Descriptive epidemiological study, Japan

## Abstract

**Objective:**

Few studies have reported intakes of fermented foods with their clear definitions. This study aimed to identify fermented foods and beverages consumed in Japan based on international definitions and to estimate their intake and contribution to energy and nutrients.

**Design:**

Data from a 16-d (four non-consecutive days within each season at 3-month intervals) semi-weighted dietary records (DR) were used. To identify ‘entirely fermented foods’ and ‘partially fermented foods’, a literature search on food processing and ingredients was conducted for all foods that appeared in the DR. For ‘partially fermented foods’, only the weight of the fermented food component was included in the estimation of total fermented food intake.

**Setting:**

Four regions in Japan: Osaka, Nagano, Tottori and Okinawa.

**Participants:**

Two-hundred forty-two apparently healthy Japanese adults aged 31–81 years.

**Results:**

Of the 1396 kinds of unique foods that appeared in the DR, 101 were ‘entirely fermented foods’ and 104 were ‘partially fermented foods’. The mean intake of fermented foods was 438 g/d per person (17 % of the total weight). They were mainly derived from beer, coffee, bread and yogurt. The mean contribution of fermented foods to the total energy intake was 18 %. For nutrients, the contribution to total intake was high to Na (46 %), Mg (22 %) and Ca (20 %).

**Conclusions:**

Fermented foods account for approximately one-fifth of the total weight and energy of dietary intake and are important contributors to some nutrients in Japanese adults.

Fermented foods, defined as ‘foods made through desired microbial growth and enzymatic conversions of food components’, have long been produced and consumed based on knowledge passed down from generation to generation in most cultures around the world^(1)^. The process of fermentation, through the action of microorganisms, improves the shelf life and flavor of foods, modifies the nutritional characteristics of foods and may even add bioactive substances that have health benefits^(2)^.

There is growing research into the health benefits of fermented foods around the world^(3–5)^. In particular, there are many reports on fermented dairy products (e.g. yogurt and cheese)^(6–10)^. For example, observational studies have reported that yogurt consumption is associated with a lower risk of gastroenteritis in one-year-old Japanese children^(6)^, and consumption of fermented dairy products is associated with a lower risk of depression in middle-aged Finnish men^(8)^. Several meta-analyses have also reported associations with lower risk of CVD^(7)^, type 2 diabetes^(9)^ and cancer^(10)^. In addition, fermented soya products have also received attention in recent years. It has been reported in observational studies in Japanese adults that natto and miso consumption is associated with a lower risk of hypertension^(11)^, arteriosclerosis^(12)^ and total mortality^(13)^. There are also various observational and intervention studies identifying the health effects of consumption of fermented food (in general). For example, it has been reported to be associated with improved immune status^(14)^, lower prevalence of atopic dermatitis^(15)^ and lower risk of mental health problems^(16)^ in adults. In addition, maternal fermented food consumption during pregnancy is associated with a lower risk of premature birth^(17)^ and a lower risk of sleep duration in their infants^(18,19)^.

Although previous studies have identified several foods as ‘fermented foods’ and discussed their health benefits, most of them are not clear on the definition of ‘fermented foods’ and the details of how to identify them. Additionally, previous studies have often used the FFQ with a limited number of foods as a dietary assessment method, suggesting that they may not have been able to cover all or even the major ‘fermented foods’ consumed in the target population. There was a study conducted in the Netherlands that estimated the intake of fermented foods, but this study had several limitations^(20)^: the definition of ‘fermented food’ in their study was unclear, did not describe how to identify fermented foods and did not provide a detailed estimate of the intake of composite foods (partially fermented foods), which are a mixture of fermented and non-fermented foods.

Therefore, observational (including descriptive) epidemiological studies examining ‘fermented foods’ should include all or most of them consumed by the target population with their clear definitions. In addition, due to differences in food culture, it has been reported that the fermented foods consumed differ between East Asian compared with Western countries. For example, breads, fermented dairy products, and fermented meat products are commonly consumed in Western countries, whereas fermented vegetable products, fermented soya products, and fermented fish products are commonly consumed in East Asian countries^(21)^. For this reason, the identification of fermented foods should be conducted for each population with similar food cultures.

The aim of this study was to clearly identify fermented foods and beverages consumed in Japan based on international definitions and to estimate their intake and contribution to energy and major nutrients, to provide a basis for future nutritional epidemiological studies on fermented foods.

## Materials and methods

### Participants

This study was based on data previously collected between November 2002 and September 2003 in four regions of Japan with large differences in geographic areas and dietary habits: Osaka (Osaka City, urban), Okinawa (Ginowan City, urban island), Nagano (Matsumoto City, rural inland) and Tottori (Kurayoshi City, rural coastal). The details of this study have been provided elsewhere^(22,23)^.

Briefly, in each of the four regions, participants were recruited by registered dietitians. Eight healthy married couples were recruited in each 10-year age band (30–39, 40–49, 50–59 and 60–69 years). The inclusion criteria for this study were the lack of self-report of major chronic diseases (such as diabetes and CVD), as well as community-dwelling (free-living) individuals. Dietitians, those who had received dietary counseling from a doctor or dietitian and those who had a history of hospitalisation for diabetes education were excluded from the study. In total, 256 adults (128 men and 128 women) were recruited. However, ten participants (five men and five women) were excluded because of missing or problematic responses on dietary records or characteristics, and four participants (two men and two women) were excluded because they had only eight days of dietary record data.

### Dietary assessment

The participants completed 4-d semi-weighted dietary records four times with 3-month intervals (total in 16 d): November and December 2002 in autumn, February 2003 in winter, May 2003 in spring and August and September 2003 in summer. The four recording days consisted of three weekdays (Monday through Friday) and one weekend day (Saturday and Sunday), which were randomly selected within approximately two weeks.

Registered dietitians in the regional centre provided the participants with written and verbal directions on maintaining dietary records and provided them with a sample-completed record as an example. Each pair was provided with recording sheets and a KD-173 digital scale (Tanita, Tokyo, Japan; precision ± 2 g at 0–250 g and ± 4 g at 251–1000 g) and was instructed on how each food and drink should be weighed. They were requested to document and weigh all foods and drinks consumed on each recording day. On occasions when weighing was problematic (e.g. dining out), they were instructed to document as much information as possible, including the brand name of the food, consumed portion size (based on typical household measures) and details of leftovers. Then, the regional centre staff reviewed the record forms and, when necessary, called or faxed for additional information or corrections to the records. All records collected were reviewed by trained registered dietitians at each regional and research centre.

As requested by the study protocol, portion sizes estimated using household measures were converted into weights in grams, and individual food items were coded based on the Standard Tables of Food Composition in Japan^(24)^.

### Identification of fermented foods and beverages consumed by Japanese adults

In this study, the data from the 16-d dietary records were used to identify the following two food groups: ‘foods consisting only of fermented ingredients (entirely fermented foods)’ and ‘foods containing fermented and non-fermented ingredients (partially fermented foods)’. This identification is based on the International Scientific Association for Probiotics and Prebiotics definition of ‘foods made through desired microbial growth and enzymatic conversions of food components’^(1)^.

#### Identification and categorisation of ‘entirely fermented foods’ and ‘partially fermented foods’

**Fig. 1:**
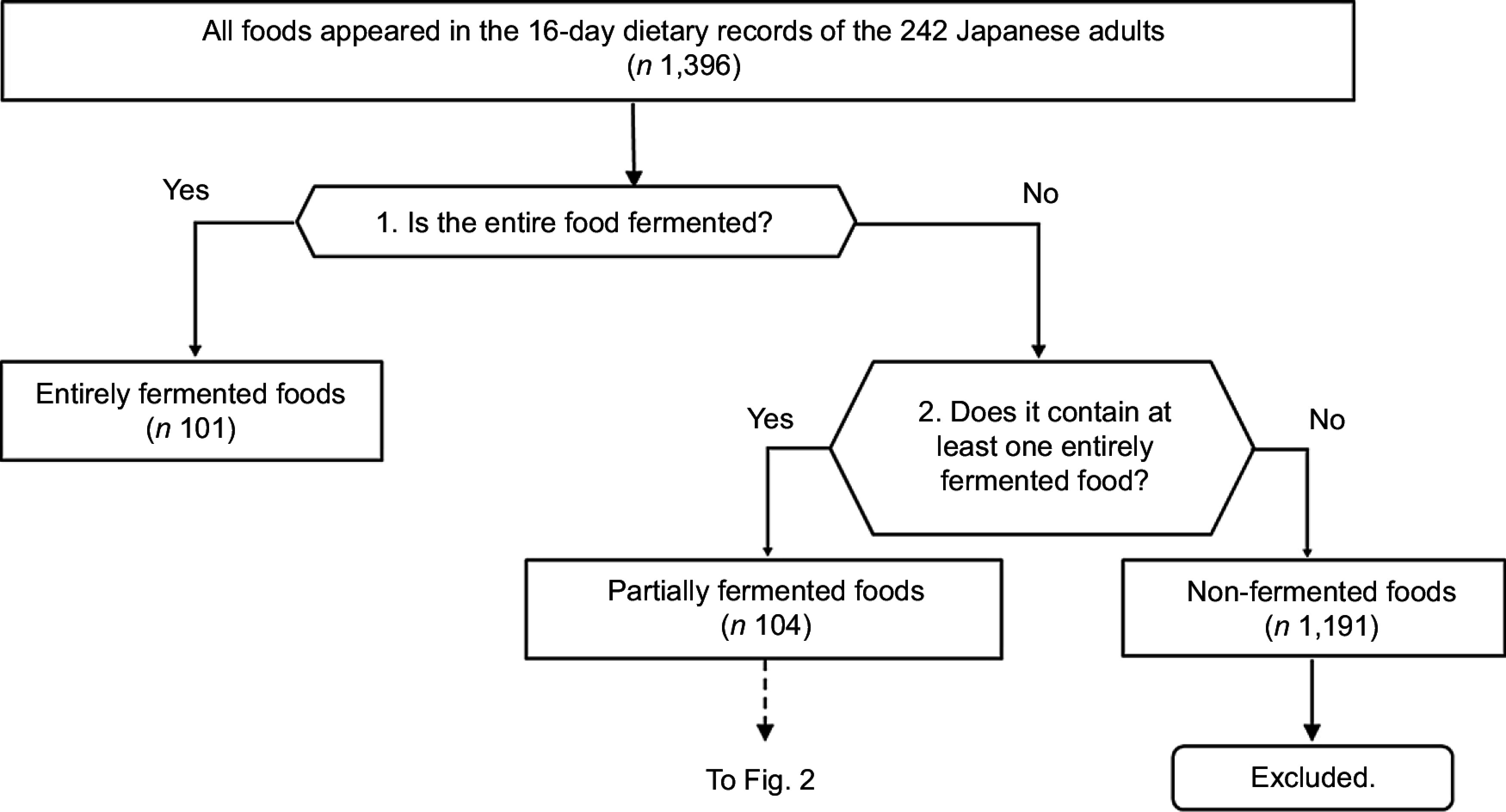
Flow chart of the procedure for identification of entirely fermented foods and partially fermented foods

The details of the procedure for identifying ‘entirely fermented foods’ and ‘partially fermented foods’ are as follows (Fig.1).

1. We examined the details of the food production process of the food that appeared in the dietary records to ensure that the entire food met the International Scientific Association for Probiotics and Prebiotics definition^(1)^. The details of the food production process were confirmed using the following procedure: First, we checked whether the description of the food listed in the Standard Tables of Food Composition in Japan 2010 Edition – Fifth Revised Edition^(24)^ indicated that the food was produced through fermentation. Second, we searched the academic literature in English or Japanese using Google Scholar to confirm the production method. Third, we referred to the manufacturer’s website to determine whether the food was fermented.
2. Of the foods not entirely fermented, composite foods that contained one or more entirely fermented foods were identified as ‘partially fermented foods’. The determination of whether the food contained one or more entirely fermented foods was made by referring to the Standard Tables of Food Composition in Japan^(24)^, academic literature or the manufacturer’s website.

As an exception to the identification of ‘entirely fermented foods’, foods such as processed cheese, which is a mixture of several ‘entirely fermented foods’ after fermentation, are also included in ‘entirely fermented foods’. In addition, this study excluded foods that are often described as ‘fermented foods’ but are mostly the product of non-microbial enzymatic processes, such as black tea and oolong tea, from ‘entirely fermented foods’.

Next, fermented foods were categorised in two different ways: (I) by 18 food groups and (II) by 26 food groups. The process is described below and shown in Table 2.

**Table 1:** Basic characteristics of the participants of the 16-d dietary records

	Total (*n* 242)		Men (*n* 121)		Women (*n* 121)	
	Mean	sd	Mean	sd	Mean	sd
Age (years)	51·0	11·8	52·4	12·2	49·6	11·3
Body height (cm)	161·0	9·0	167·4	6·6	154·7	6·2
Body weight (kg)	59·7	11·5	66·4	10·4	53·0	8·2
BMI (kg/m^2^)	22·9	3·2	23·6	2·9	22·2	3·3

SD, standard deviation; BMI, body mass index.

**Table 2:** Number of fermented foods appeared in the DR by food group in the two categories

	Methods of categorisation	Name of food group	*n*
Category I	Foods were categorised into eighteen food groups according to the Standard Tables of Food Composition in Japan.	Cereals	16
		Potatoes and starches	0
		Sugars and sweeteners	0
		Pulses	7
		Nuts and seeds	0
		Vegetables	29
		Fruits	2
		Mushrooms	1
		Algae	4
		Fish, mollusks and crustaceans	33
		Meat	6
		Eggs	3
		Milk and milk products	15
		Fats and oils	0
		Confectionaries	21
		Beverages	28
		Seasonings and spices	39
		Prepared foods	1
			
Category II	Independent of Category I, foods were categorised into twenty-six food groups by focusing on ‘entirely fermented food(s)’ included in each food. When one food included more than one ‘entirely fermented food’, the food was categorised into more than one food group. Therefore, the total number of foods in Category II was larger than the total number of foods in Category I.	Breads	22
		Rice-koji (fermented rice grains)	3
		Flour confectionery	5
		Natto (fermented soybean)	3
		Other soya products	3
		Pickles	17
		Katsuo-bushi (dried bonito)	8
		Shiokara	3
		Processed meats	3
		Yogurt	3
		Lactic acid bacteria beverages	3
		Cheese	9
		Sake (Japanese rice wine)	16
		Beer	2
		Wine	4
		Coffee	4
		Cocoa	4
		Mirin (sweet sake)	20
		Spirits	20
		Other beverages	2
		Soya sauce	53
		Vinegar	25
		Miso (fermented soybean paste)	10
		Sakekasu (sake lees)	3
		Other seasonings	4
		Yeast	1

DR, dietary records.

In Category I, foods were categorised into eighteen food groups (cereals, potatoes and starches, sugars and sweeteners, pulses, nuts and seeds, vegetables, fruits, mushrooms, algae, fish, meat, eggs, milk and milk products, fats and oils, confectionaries, beverages, seasonings and spices and prepared foods) based on the food groups of the Standard Tables of Food Composition in Japan^(24)^. For example, pickled cucumber with seasoned vinegar (food code: 6069) was categorised in the ‘vegetables’ food group.

In Category II, independent of Category I, foods were categorised into twenty-six food groups by focusing on ‘entirely fermented food(s)’ included in each food: breads, rice-koji (fermented rice grains), flour confectionery, natto (fermented soyabean), other soya products, pickles, katsuo-bushi (dried bonito), shiokara (salted fish), processed meats, yogurt, lactic acid bacteria beverages, cheese, sake (japanese rice wine), beer, wine, coffee, cocoa, mirin (sweet sake), spirits, other beverages, soya sauce, vinegar, miso (fermented soybean paste), sakekasu (sake lees), other seasonings and yeast. For example, pickled cucumber with seasoned vinegar (food code: 6069) was categorised in the ‘vinegar’ food group. When one food included more than one ‘entirely fermented food’, the food was categorised into more than one food group. Therefore, the total number of foods in Category II was larger than the total number of foods in Category I.

The list of foods identified as ‘entirely fermented foods’ and ‘partially fermented foods’ which were used to estimate the intake of fermented foods in this study is shown in online supplementary material, Table S1 with their characteristics.

#### Determination of the weight proportion of fermented foods to ‘partially fermented foods’

**Fig. 2:**
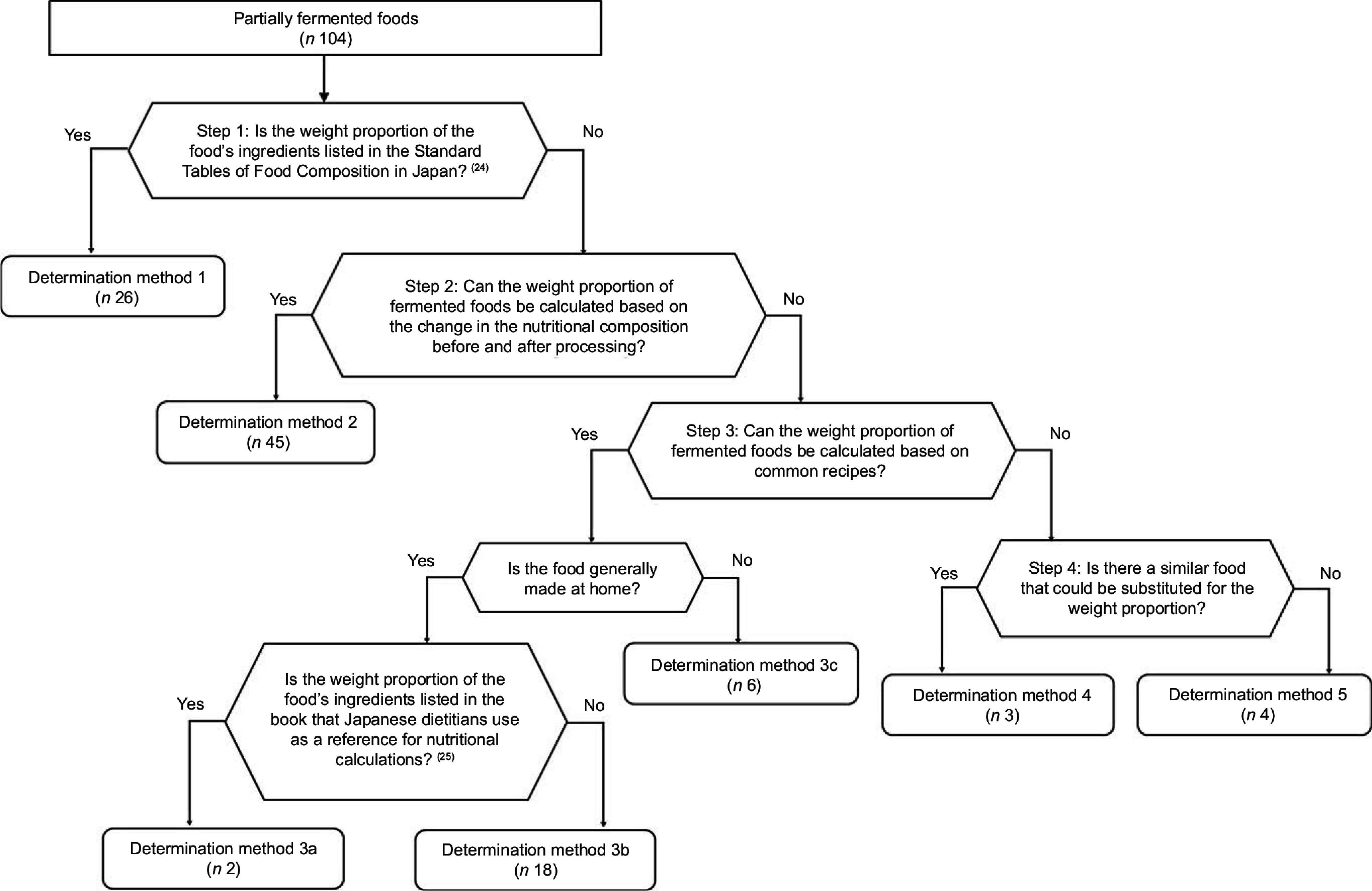
Flow chart of the procedure for determination of the weight proportion of fermented foods for partially fermented foods

To estimate the intake of fermented foods derived from ‘partially fermented foods’, the weight proportion of fermented foods was determined for each food. This was determined using the following steps and shown in Fig.2. Steps are sequential, meaning if one step fails to identify the proportion of partially fermented food, we would move on to the next step.

1. For foods that reported the proportion of ingredients on the ‘Notes by Food Groups’ in the Standard Tables of Food Composition in Japan^(24)^, we calculated the weight proportion of fermented foods by using this information (Determination method 1).
2. For foods that the weight proportion of fermented foods could be calculated based on the change in the nutritional composition before and after processing, we determined the proportion based on the nutritional composition. For example, the proportion of vinegar was calculated based on the change in acetic acid content by food processing (Determination method 2).
3. If the weight proportion of fermented foods could be calculated based on common recipes, the following steps were taken. For foods that could be made at home, we first checked a book on culinary science that Japanese registered dietitians often refer to for calculation of nutritional values^(25)^. (a) For foods for which their recipes (proportions of ingredients) were listed, we determined their weight proportions based on the recipes (Determination method 3a). (b) For foods that were not listed in the book, we determined their weight proportions based on the mean of the three recipes arbitrarily selected from recipes found on the cooking websites (Determination method 3b). (c) For foods that are less likely to be made at home, we determined their weight proportions based on information on ingredients found on the websites of manufacturers with a large share of sales (Determination method 3c).
4. For foods with the similar food, we used the values of foods with similar ingredients (Determination method 4). For foods without the similar food, we estimated the weight proportions from the names of the ingredients (Determination method 5).

### Other measurements

Body height was measured to the nearest 0·1 cm. Body weight was measured using light clothing to the nearest 0·1 kg. BMI (kg/m^2^) was calculated as body weight (kg) divided by the square of the body height (m).

### Statistical analysis

To describe the intake of fermented foods in the targeted population, we calculated the percentage of participants who ate the foods at least once during the study period. We also calculated the mean and sd of the mean intake per participant per day and the median of the median daily intake per participant per day. The total intake of fermented foods is defined as the sum of the ‘entirely fermented foods’ and the fermented food parts of ‘partially fermented foods’. The intake for each entirely fermented food group (in Category II) is the sum of the intake of ‘entirely fermented foods’ and the intake of entirely fermented foods derived from ‘partially fermented foods’.

In addition, energy, water and thirty-one selected nutrient intakes from fermented foods were estimated. The mean contribution of fermented foods to total food intake (%) in the population was calculated as follows. First, the contribution of each nutrient to total intake for each participant was calculated for every day of recording. Then, using these values, a mean of the 16 d of recording for each participant was calculated. Alcohol was excluded from the analysis because all alcohol is produced by fermentation although its health effect should be considered. For the calculations of energy and nutrients, we used the Standard Tables of Food Composition in Japan^(24)^. All statistical analyses were performed using the SAS statistical software (version 9·4, SAS Institute Inc.).

## Results

The present analysis included 242 Japanese adults (121 women and 121 men) with a mean age of 51·0 years (49·6 years for women and 52·4 years for men). The participant characteristics are shown in Table 1.

With each participant providing 16 d of dietary intake, there was a total of 3872 d of dietary records. A total of 1396 kinds of unique foods were reported to be consumed during the study period, of which 101 were ‘entirely fermented foods’, 104 were ‘partially fermented foods’ and 1191 were ‘non-fermented foods’.

Table 2 shows the number of foods by food group in each of the two categories. In Category I, seasonings were the most common fermented food, followed by fish, vegetable, beverages and confectionaries. In Category II, soya sauce was the most common fermented food, followed by vinegar, breads, mirin (sweet sake) and spirits.

Table 3 shows the mean (sd) and median of food intakes (g/d per person) by fermentation (entirely, partially, or none). The mean total weight of food reported to be consumed was 2573·9 g/d per person. On average, ‘entirely fermented foods’ made up 16·1 % of total intake (416·5 g/d per person) and ‘partially fermented foods’ made up 0·9 % (22·4 g/d per person). Overall, the mean intake of fermented foods ‘entirely fermented foods’ plus ‘partially fermented foods’ was 17·1 % of total food intake (437·9 g/d per person).

**Table 3:** Mean (sd) and median of food intakes (g/d per person) by fermentation (entirely, partially, or none)

Category of fermentation	Mean	sd	Median
All foods appeared in the dietary records (*n* 1396*)	2573·9	606·9	2446·9
Entirely fermented foods (*n* 101)	416·5	305·1	294·7
Partially fermented foods (*n* 104)	133·9	81·4	83·7
(Fermented food parts of partially fermented foods (*n* 104))	(22·4)	(16·7)	(8·3)
Total fermented foods† (*n* 205)	437·9	306·0	315·7
Non-fermented foods (*n* 1191)	2136·3	516·4	2092·2

SD, standard deviation.

* Number of foods included in the category.

† Sum of the entirely fermented foods and the fermented food parts of partially fermented foods.

Table 4 shows the percentage of participants who consumed each of the twenty-six entirely fermented food groups in Category II at least once during the survey period and the amount of intake per person per day for each food group. The entirely fermented food groups in Category II with a high frequency of intake during the survey period were katsuo-bushi (bonito flakes), mirin, spirits, soya sauce, vinegar and miso (100 %), followed by breads (98 %), sake and coffee (95 %). The groups with highest mean intake were, in this order, beer (140·5 g/d per person), coffee (114·3 g/d per person), bread (40·9 g/d per person), yogurt (33·2 g/d per person) and soya sauce (20·3 g/d per person).

**Table 4:** Descriptive statistics on intakes of the twenty-six entirely fermented food groups in category II

Entirely fermented food groups consumed by Japanese adults†	Percentage of participants who consumed each of the food groups at least once during the survey period (%).	Intakes (g/person per d)*		
		Mean	sd	Median
Breads (*n* 22‡)	98	40·9	28·7	16·4
Rice-koji (fermented rice grains) (*n* 3)	10	0·1	0·3	0·0
Flour confectionery (*n* 5)	28	1·0	2·5	0·0
Natto (fermented soybean) (*n* 3)	70	6·5	7·9	0·0
Other soya products (*n* 3)	6	0·1	0·3	0·0
Pickles (*n* 107)	85	8·2	10·1	0·0
Katsuo-bushi (dried bonito) (*n* 8)	100	1·1	1·1	0·6
Shiokara (*n* 3)	8	0·3	1·2	0·0
Processed meats (*n* 3)	17	0·2	0·7	0·0
Yogurt (*n* 3)	74	33·2	42·6	0·0
Lactic acid bacteria beverages (*n* 3)	31	4·9	11·7	0·0
Cheese (*n* 9)	77	3·3	3·9	0·0
Sake (Japanese rice wine) (*n* 16)	95	14·7	39·7	0·0
Beer (*n* 2)	62	140·5	241·9	0·0
Wine (*n* 4)	40	7·7	31·4	0·0
Coffee (*n* 4)	95	114·3	137·8	6·3
Cocoa (*n* 4)	68	0·5	0·8	0·0
Mirin (sweet sake) (*n* 20)	100	4·6	3·3	0·6
Spirits (*n* 20)	100	16·2	48·8	0·0
Other beverages (*n* 2)	4	0·5	3·2	0·0
Soya sauce (*n* 53)	100	20·3	7·8	17·7
Vinegar (*n* 25)	100	7·5	6·1	4·1
Miso (fermented soybean paste) (*n* 10)	100	10·8	5·9	10·0
Sakekasu (sake lees) (*n* 3)	27	0·3	0·6	0·0
Other seasonings (*n* 4)	45	0·2	0·5	0·0
Yeast (*n* 1)	5	0·0	0·2	0·0

SD, standard deviation.

* Calculated from the sum of the entirely fermented foods and fermented food parts of partially fermented foods.

† Details of the foods categorised in each ‘entirely fermented foods’ are shown in see online supplementary material, Table S1.

‡ Number of foods included in the food group.

Table 5 shows the mean (sd) and percentiles of energy, water and thirty-one selected nutrients derived from fermented foods and their contributions to total intake. The mean energy intake from fermented foods was 394 kcal/person per d (18·2 % of the mean total energy intake). The nutrients to which fermented foods highly contributed were, in this order, Na (46·1 %, 2053·0 mg/person per d), Mg (22·4 %, 63·8 mg/person per d), Ca (19·8 %, 113·5 mg/person per d), niacin (17·9 %, 3·2 mg/person per d) and soluble dietary fibre (17.5 %, 0.6 g/person per d).

**Table 5:** Mean (sd) and percentiles of energy, water and thirty-one selected nutrients derived from fermented foods* and their contributions to total intake

		Daily intakes (person/d)							Contribution of fermented foods* to total intake (%) †	
	Unit	Mean	sd	Percentile					Mean	sd
				5 %	25 %	Median	75 %	95 %		
Energy	kcal	394	255	135	239	313	477	877	18·2	9·0
Water	g	353	272	81	160	266	468	864	15·2	9·8
Carbohydrate	g	39·7	17·1	14·2	28·3	38·5	47·2	72·0	14·7	6·2
Total dietary fibre	g	2·3	1·0	0·9	1·7	2·2	2·8	4·1	16·1	6·3
Soluble dietary fibre	g	0·6	0·3	0·2	0·4	0·5	0·7	1·0	17·5	7·3
Insoluble dietary fibre	g	1·7	0·7	0·7	1·2	1·6	2·2	3·0	16·9	6·6
Protein	g	11·9	3·9	6·5	9·1	11·4	14·1	19·7	15·9	4·8
Total fat	g	5·2	2·5	1·7	3·4	4·7	6·5	10·0	8·9	4·1
Saturated fat	g	1·8	1·1	0·4	1·0	1·6	2·4	3·9	10·8	6·1
Monounsaturated fat	g	1·3	0·7	0·4	0·8	1·2	1·7	2·5	6·8	3·4
Polyunsaturated fat‡	g	1·2	0·6	0·5	0·8	1·1	1·5	2·4	9·8	4·4
Cholesterol	mg	8·9	7·0	1·1	3·6	6·8	12·6	21·9	3·4	2·8
Na	mg	2053·0	620·4	1155·5	1624·6	1965·0	2483·9	2985·4	46·1	8·0
Potassium	mg	457·3	160·4	236·7	340·7	431·5	562·8	753·8	16·9	5·3
Ca	mg	113·5	62·7	40·4	67·4	95·8	147·7	238·3	19·8	7·9
Mg	mg	63·8	19·2	36·1	50·9	60·5	75·2	97·8	22·4	5·9
Phosphorus	mg	197·5	70·4	98·4	146·3	188·1	231·7	330·7	17·2	4·8
Fe	mg	1·6	0·5	0·9	1·3	1·5	1·9	2·6	0·7	0·3
Zn	mg	1·2	0·5	0·6	0·9	1·2	1·4	2·0	14·1	4·7
Cu	mg	0·2	0·1	0·1	0·1	0·2	0·2	0·3	14·3	5·2
Manganese	mg	0·4	0·3	0·1	0·2	0·3	0·5	1·0	12·1	10·8
Vitamin A retinol equivalent§	*μ*g	20·6	19·0	0·6	6·2	15·2	30·7	56·1	4·4	3·8
Vitamin D	*μ*g	0·1	0·6	0·0	0·0	0·0	0·1	0·1	1·6	1·9
*α*-tocopherol	mg	0·4	0·2	0·2	0·3	0·4	0·5	0·8	7·1	2·8
Vitamin K	*μ*g	47·0	54·6	0·9	7·3	26·4	67·9	173·9	12·3	11·5
Thiamin (vitamin B_1_)	mg	0·1	0·0	0·0	0·1	0·1	0·1	0·1	9·2	4·3
Riboflavin (vitamin B_2_)	mg	0·2	0·1	0·1	0·1	0·2	0·2	0·4	14·9	5·5
Niacin	mg	3·2	1·4	1·4	2·2	3·0	3·7	6·3	17·9	7·5
Vitamin B_6_	mg	0·2	0·2	0·1	0·1	0·2	0·2	0·7	16·6	11·9
Vitamin B_12_	*μ*g	0·5	0·3	0·1	0·3	0·4	0·5	1·0	8·6	5·2
Folate	*μ*g	47·0	17·9	21·5	34·9	44·1	54·6	84·6	14·0	5·1
Pantothenic acid	mg	0·9	0·4	0·3	0·6	0·8	1·1	1·8	13·6	5·4
Vitamin C	mg	1·7	2·1	0·0	0·3	1·1	2·3	5·8	1·7	2·0

SD, standard deviation.

* Calculated from the sum of the entirely fermented foods and the fermented food parts of partially fermented foods.

† The mean contribution of the population was calculated as follows: the contribution of each subject was calculated for each day, and the mean contribution of each subject for 16 d was calculated, then the mean contribution of the population was calculated using the means of each subject.

‡ Sum of eicosapentaenoic acid, docosapentaenoic acid and DHA.

§ Sum of retinol, *β*-carotene/12, *α*-carotene/24 and cryptoxanthin/24.

## Discussion

To our knowledge, this is the first study to identify ‘fermented foods’ under a clear definition and procedure, to develop a method to estimate the total intake of fermented foods, taking into account indirect intake from ‘partially fermented foods’, and to show the intake of energy and nutrients derived from fermented foods and their contribution to the total diet.

In this study, we focused on the foods consumed by the Japanese adult population for use in future nutritional epidemiological studies. Of the 1396 foods consumed by the participants in this study, 101 were identified as ‘entirely fermented foods’ consisting of fermented foods only, 104 as ‘partially fermented foods’ containing fermented foods in parts. This study revealed that fermented foods accounted for 17 % of the total dietary intake of Japanese adults. The major food groups with the highest intake were beverages, dairy products and cereals/grains. Among the twenty-six entirely fermented food groups (Category II in Tables 2 and 4), beer, coffee, bread and yogurt were consumed the highest, in this order. They were foods with high water content (e.g. beverages). This result was similar to the previous study in the Netherlands. However, soya sauce followed to yogurt. Miso was in the eighth order. These were typical Japanese seasonings used in relatively small quantities. Foods that did not appear at the top of the list in terms of consumption by weight due to the small portion size per serving were katsuo-bushi (bonito flakes), mirin (sweet cooking rice wine), vinegar and miso (soybean paste). These are also fermented foods popular in Japan as evidenced by 100 % of the study population consuming these foods.

We also found that in this Japanese adult population, 18 % of total energy is derived from fermented foods. Among the nutrients, 46 % of Na, 22 % of Mg 20 % of Ca, 18 % of niacin and soluble dietary fibre were consumed from fermented foods. These results clearly indicate that fermented foods are an important food group in nutritional epidemiology.

The reason why Na was the major nutrient consumed from fermented foods was probably due to the fact that salt is often added during the production process of fermented foods. This trend was also reported in a previous study conducted in South Korea, an Asian country, as well as Japan^(26)^. The fermented foods with highest contribution to Na intake in this study were soya sauce (26·2 % of total intake), miso (11·5 %) and bread (4·6 %). High Na intake is a known risk factor for hypertension, and reducing salt intake can prevent hypertension^(27,28)^. The physiological requirement for Na in adults is < 1 g/d^(29)^, and the WHO recommends a maximum dietary Na intake of 2 g (5 g salt)^(30)^. However, the results of Na intake also indicate that Japanese adults from this study are consuming their daily maximum just from fermented foods, and that their total intake is well above WHO recommendations.

Some of the nutrients with high contributions in this study were synthesised as metabolites by microorganisms through fermentation. It has been reported that the concentration of many vitamins in foods, such as riboflavin (vitamin B_2_), folic acid, vitamin B_12_ and vitamin K, is increased by fermentation^(4)^. The contributions of this study were 14·9 % for riboflavin (vitamin B_2_), 14·0 % for folate, 8·6 % for vitamin B_12_ and 12·3 % for vitamin K. In particular, vitamin K has been reported to be abundant in fermented foods, especially in natto^(31)^. Natto is a traditional Japanese fermented soybean product that contributed to most of the fermented food-derived vitamin K intake examined in this Japanese adult population (87·5 % contribution to vitamin K intake derived from total fermented foods).

All alcoholic beverages are produced by fermentation. Excessive consumption of alcohol is a risk for both mental and physical health problems^(32)^. Na intake from fermented foods was high (46 % of all the Na intake). Therefore, although fermented foods have received attention for their positive health effects^(3–5)^, we should consider possible unfavourable nutrients or substances contained in some fermented foods such as Na and alcohol when we examine the health effects of fermented foods.

The strength of our study is that it used data from 16-d dietary records conducted in four regions of Japan with different characteristics to comprehensively identify fermented foods among the foods consumed by the target population, with clear definitions and procedures. In addition, in order to estimate more accurately the total intake of fermented foods, the indirect intake of fermented foods derived from ‘partially fermented foods’ as also included after determining the weight proportion of fermented foods contained in ‘partially fermented foods’. This has not been conducted in previous studies.

However, this study has several limitations. First, the dietary record data used in this study were coded based on the Standard Tables of Food Composition in Japan; therefore, the number of foods that included in this study may be less than the number of foods actually consumed by this population. Second, some of the foods identified in this study as ‘entirely fermented foods’ were traditionally made through a fermentation process, but due to the diversification of production processes in recent years, some of them may not have gone through the fermentation process. Because the dietary record data used in this study lacked information to determine whether the food was made through the process of fermentation, we decided to include all foods that were likely to have been fermented as a result of the literature search. Therefore, fermented food intake may have been overestimated compared with actual intake. Third, there was limited information available in the literature research conducted to find information on which to base the determination of the weight proportion of fermented foods of ‘partially fermented foods’, and the composition differed among products and recipes, even if they belonged to the same food name. Fourth, the data used in this study were collected from 2002 to 2003; therefore, the current dietary intake of the Japanese population may have differed from that in this study. For example, a study that investigated dietary pattern trends among Japanese adults over a 13-year period from 2003 to 2015 using data from the National Health and Nutrition Survey reported that the intake of bread and dairy, animal foods and oils increased, whereas the intake of plant foods and fish decreased^(33)^. Fifth, the 16-d dietary records were obtained from cohabiting couples, which may have reduced the variety of foods consumed by the target population and inter-individual variation in dietary intake. Sixth, the participants were not randomly selected and may not be representative of the general Japanese population. The survey areas were not equally distributed across the country, but were located mostly in the western parts of Japan. Therefore, the generalisability of the results may be limited. Nevertheless, the participants’ weights and heights were similar to those of the general Japanese population^(34)^.

### Conclusion

We identified fermented foods consumed in Japan using clear definitions and procedures. Fermented foods account for approximately one-fifth of the total weight and energy of dietary intake and are important contributors to some nutrients in Japanese adults. This study provides a foundation for future nutritional epidemiological studies of fermented foods.

## Supporting information

Fujihashi et al. supplementary material Fujihashi et al. supplementary material

## Supplementary material

For supplementary material accompanying this paper visit https://doi.org/10.1017/S1368980024000405.
