# Supplementary material for: Identification and estimation of the intake of fermented foods and their contribution to energy and nutrients among Japanese adults
Source: Public Health Nutr. 2024 Feb 16;27(1):e153. doi: 10.1017/S1368980024000405 (PMC11617413; doi:10.1017/S1368980024000405)
Supplement: Fujihashi et al. supplementary material [file S1368980024000405sup001.docx]

**Table S1.** Characteristics of the fermented foods included in this study and the mean intake of each food

| Food code in the STFCJ^(24)^ | Food name in the STFCJ^(24)^ | Category I | Category II | For partially fermented foods | | | | | Total number of appeara-nces | Mean intake (g/day)* | Mean intake (g/time)† |
| --- | --- | --- | --- | --- | --- | --- | --- | --- | --- | --- | --- |
|  |  |  |  | Contained entirely fermented foods | | The weight proportion of fermented foods [fermented foods(g)/total weight(g)] | | |  |  |  |
|  |  |  |  | Food code | Food name | For each food | Total | Calcula-tion Method |  |  |  |
| Entirely fermented foods | |  |  |  |  |  |  |  |  |  |  |
| 1026 | Common wheat, bread, white | Cereals | Breads | - | - | - | - | - | 1423 | 26.4 | 71.8 |
| 1028 | Common wheat, bread, white long roll | Cereals | Breads | - | - | - | - | - | 145 | 2.2 | 57.8 |
| 1030 | Common wheat, bread, hardtack | Cereals | Breads | - | - | - | - | - | 1 | 0.0 | 60.0 |
| 1031 | Common wheat, bread, French bread | Cereals | Breads | - | - | - | - | - | 83 | 1.2 | 53.8 |
| 1032 | Common wheat, bread, rye and wheat bread | Cereals | Breads | - | - | - | - | - | 43 | 0.7 | 60.7 |
| 1034 | Common wheat, bread, soft rolls | Cereals | Breads | - | - | - | - | - | 168 | 2.7 | 63.3 |
| 1036 | Common wheat, bread, English muffins | Cereals | Breads | - | - | - | - | - | 15 | 0.3 | 75.5 |
| 1037 | Common wheat, bread, naan | Cereals | Breads | - | - | - | - | - | 7 | 0.1 | 71.4 |
| 1076 | Common wheat, pizza crust | Cereals | Breads | - | - | - | - | - | 30 | 0.4 | 56.1 |
| 1077 | Common wheat, bread crumbs, fresh | Cereals | Breads | - | - | - | - | - | 27 | 0.1 | 11.3 |
| 1078 | Common wheat, bread crumbs, semi-dry | Cereals | Breads | - | - | - | - | - | 12 | 0.0 | 8.5 |
| 1079 | Common wheat, bread crumbs, dry | Cereals | Breads | - | - | - | - | - | 987 | 1.0 | 4.0 |
| 1116 | Rice, non-glutinous rice products, rice-koji | Cereals | Rice-koji | - | - | - | - | - | 10 | 0.0 | 12.3 |
| 4043 | Soybeans, tofu, "Tofu-yo" (fermented tofu) | Pulses | Other soy products | - | - | - | - | - | 4 | 0.0 | 10.0 |
| 4046 | Soybeans, natto, "Itohiki-natto" (fermented whole soybean) | Pulses | Natto | - | - | - | - | - | 641 | 5.7 | 34.4 |
| 4047 | Soybeans, natto, "Hikiwari-natto" (fermented dehulled and split soybean) | Pulses | Natto | - | - | - | - | - | 71 | 0.8 | 41.0 |
| 4048 | Soybeans, natto, "Goto-natto" (natto fermented with rice-koji and salt) | Pulses | Natto | - | - | - | - | - | 1 | 0.0 | 43.0 |
| 4061 | Soybeans, "Kinzanji-miso" (fermented roasted soybeans, barley or wheat, vegetable and salt with koji mold) | Pulses | Other soy products | - | - | - | - | - | 13 | 0.1 | 16.5 |
| 4062 | Soybeans, "Hishio-miso" (fermented soybeans, barley or wheat, and vegetable with koji mold) | Pulses | Other soy products | - | - | - | - | - | 2 | 0.0 | 8.0 |
| 6043 | Turnip, pickles, "Nukamiso-zuke" (pickled in salty rice bran paste), leaves | Vegetables | Pickles | - | - | - | - | - | 3 | 0.0 | 17.7 |
| 6044 | Turnip, pickles, "Nukamiso-zuke" (pickled in salty rice bran paste), root with skin | Vegetables | Pickles | - | - | - | - | - | 9 | 0.0 | 19.7 |
| 6068 | Cucumber, fruit, pickles, "Nukamiso-zuke" (pickled in salty rice bran paste) | Vegetables | Pickles | - | - | - | - | - | 69 | 0.4 | 23.2 |
| 6070 | Cucumber, fruit, pickles, sour type (processed by lactic acid fermentation) | Vegetables | Pickles | - | - | - | - | - | 11 | 0.1 | 25.5 |
| 6088 | Stem mustard, pickles | Vegetables | Pickles | - | - | - | - | - | 21 | 0.0 | 8.1 |
| 6108 | Oriental pickling melon, fruits, pickles, "Nara-zuke" (pickled with Sake lees) | Vegetables | Pickles | - | - | - | - | - | 128 | 0.5 | 13.8 |
| 6137 | Japanese radishes, Daikon, root, pickles, "Nukamiso-zuke" (pickled in salty rice bran paste) | Vegetables | Pickles | - | - | - | - | - | 34 | 0.2 | 24.3 |
| 6139 | Japanese radishes, Daikon, root, pickles, "Takuan-zuke" (pickled with rice bran and salt), made of sun-dried Daikon | Vegetables | Pickles | - | - | - | - | - | 498 | 2.1 | 16.6 |
| 6140 | Japanese radishes, Daikon, root, pickles, "Moriguchi-zuke" (slender root cultivar pickled with Sake lees) | Vegetables | Pickles | - | - | - | - | - | 22 | 0.1 | 17.0 |
| 6148 | Leaf mustard, "Takana", leaves, salted pickles | Vegetables | Pickles | - | - | - | - | - | 36 | 0.1 | 14.2 |
| 6152 | Bamboo shoots, "Shinachiku" (boiled, fermented and salted bamboo shoots), desalted | Vegetables | Pickles | - | - | - | - | - | 99 | 0.3 | 13.0 |
| 6196 | Eggplant*, pickles, "Nukamiso-zuke" (pickled with salty rice bran paste) [*Syn. Aubergine] | Vegetables | Pickles | - | - | - | - | - | 28 | 0.2 | 22.1 |
| 6199 | Eggplant*, pickles, "Shiba-zuke" (pickled with perilla leaf, cucumber and Myoga, etc.) [*Syn. Aubergine] | Vegetables | Pickles | - | - | - | - | - | 27 | 0.1 | 11.4 |
| 6230 | Turnip green, "Nozawana", leaves, pickles, salted pickles | Vegetables | Pickles | - | - | - | - | - | 238 | 1.5 | 25.0 |
| 6236 | Heading Chinese cabbage, head, pickles, Kimchi | Vegetables | Pickles | - | - | - | - | - | 257 | 2.4 | 36.5 |
| 7037 | Olives, in brine, green | Fruits | Pickles | - | - | - | - | - | 3 | 0.0 | 5.9 |
| 10091 | Fish, skipjack tuna, processed products, "Katsuo-bushi" (boiled, smoke-dried and fermented skipjack tuna fillet) | Fish, mollusks and crustaceans | Katsuo-bushi | - | - | - | - | - | 121 | 0.0 | 1.5 |
| 10092 | Fish, skipjack tuna and frigate mackerel, processed products, "Kezuri-bushi" (shaved "Katsuo-bushi"), uncooked (cf. 10091"Katsuo-bushi") | Fish, mollusks and crustaceans | Katsuo-bushi | - | - | - | - | - | 794 | 0.4 | 1.9 |
| 10095 | Fish, skipjack tuna, processed products, "Shiokara" (salted and fermented viscera) | Fish, mollusks and crustaceans | Shiokara | - | - | - | - | - | 2 | 0.0 | 88.0 |
| 10341 | Crustacean, processed crab, "Ganzuke" (salted and fermented fiddler crabs) | Fish, mollusks and crustaceans | Shiokara | - | - | - | - | - | 1 | 0.0 | 11.0 |
| 10358 | Mollusks, processed squid, "Shiokara" (salted and fermented meat and liver) | Fish, mollusks and crustaceans | Shiokara | - | - | - | - | - | 22 | 0.2 | 40.2 |
| 11182 | Pork, ham, uncooked ham, ripened | Meat | Processed meats | - | - | - | - | - | 4 | 0.0 | 22.8 |
| 11187 | Pork, sausage, semi-dry | Meat | Processed meats | - | - | - | - | - | 42 | 0.1 | 13.5 |
| 11188 | Pork, sausage, dry | Meat | Processed meats | - | - | - | - | - | 8 | 0.0 | 17.8 |
| 13025 | Yogurt, whole milk, unsweetened | Milk and milk products | Yogurt | - | - | - | - | - | 887 | 23.7 | 103.7 |
| 13026 | Yogurt, skimmed, sweetened | Milk and milk products | Yogurt | - | - | - | - | - | 274 | 6.4 | 90.6 |
| 13027 | Yogurt, liquid, sweetened | Milk and milk products | Yogurt | - | - | - | - | - | 79 | 3.0 | 148.5 |
| 13028 | Lactic acid bacteria beverages, not pasteurized after fermentation, milk solids-nonfat ≥ 3.0% | Milk and milk products | Lactic acid bacteria beverages | - | - | - | - | - | 172 | 3.7 | 82.3 |
| 13029 | Lactic acid bacteria beverages, pasteurized after fermentation, milk solids-nonfat ≥ 3.0% | Milk and milk products | Lactic acid bacteria beverages | - | - | - | - | - | 56 | 0.6 | 42.4 |
| 13030 | Lactic acid bacteria beverages, milk solids-nonfat < 3.0% | Milk and milk products | Lactic acid bacteria beverages | - | - | - | - | - | 17 | 0.6 | 140.6 |
| 13031 | Cheese, Edam | Milk and milk products | Cheese | - | - | - | - | - | 7 | 0.0 | 22.8 |
| 13033 | Cheese, cottage | Milk and milk products | Cheese | - | - | - | - | - | 4 | 0.0 | 8.9 |
| 13034 | Cheese, Camembert | Milk and milk products | Cheese | - | - | - | - | - | 28 | 0.1 | 16.6 |
| 13035 | Cheese, cream | Milk and milk products | Cheese | - | - | - | - | - | 56 | 0.3 | 19.6 |
| 13036 | Cheese, Gouda | Milk and milk products | Cheese | - | - | - | - | - | 21 | 0.1 | 23.7 |
| 13037 | Cheese, Cheddar | Milk and milk products | Cheese | - | - | - | - | - | 12 | 0.0 | 16.1 |
| 13038 | Cheese, Parmesan | Milk and milk products | Cheese | - | - | - | - | - | 65 | 0.1 | 4.6 |
| 13040 | Cheeses, processed | Milk and milk products | Cheese | - | - | - | - | - | 559 | 2.6 | 18.1 |
| 15045 | Traditional confectionery, "Karinto, black" (crunchy deep fried wheat flour dough coated with brown sugar" | Confectionaries | Flour confectionery | - | - | - | - | - | 66 | 0.5 | 27.1 |
| 15046 | Traditional confectionery, "Karinto, white" (crunchy deep fried wheat flour dough coated with white sugar) | Confectionaries | Flour confectionery | - | - | - | - | - | 4 | 0.0 | 23.8 |
| 15077 | Cake and pastry, doughnuts, yeast-leavened | Confectionaries | Flour confectionery | - | - | - | - | - | 22 | 0.3 | 44.7 |
| 15093 | Biscuits, crackers, oil-sprayed | Confectionaries | Flour confectionery | - | - | - | - | - | 49 | 0.2 | 18.4 |
| 15094 | Biscuits, crackers, soda | Confectionaries | Flour confectionery | - | - | - | - | - | 9 | 0.0 | 10.8 |
| 16001 | Fermented alcoholic beverage, "Sake", regular | Beverages | Sake | - | - | - | - | - | 1594 | 12.9 | 31.3 |
| 16002 | Fermented alcoholic beverage, "Sake", "Junmai" (made with only rice, water and koji) | Beverages | Sake | - | - | - | - | - | 4 | 0.0 | 16.3 |
| 16003 | Fermented alcoholic beverage, "Sake", "Honjozo" (made with rice, water, koji and distilled alcohol. The rice used must be polished to at least 70%) | Beverages | Sake | - | - | - | - | - | 76 | 1.1 | 56.7 |
| 16004 | Fermented alcoholic beverage, "Sake", "Ginjo" (brewed with labor-intensive steps, using highly polished rice） | Beverages | Sake | - | - | - | - | - | 10 | 0.5 | 181.3 |
| 16006 | Fermented alcoholic beverage, beer, pale | Beverages | Beer | - | - | - | - | - | 707 | 86.3 | 472.6 |
| 16009 | Fermented alcoholic beverage, "Happoshu" (beer-like beverage with less than 67% malt content) | Beverages | Beer | - | - | - | - | - | 442 | 54.0 | 472.8 |
| 16010 | Fermented alcoholic beverage, wine, white | Beverages | Wine | - | - | - | - | - | 93 | 2.1 | 89.4 |
| 16011 | Fermented alcoholic beverage, wine, red | Beverages | Wine | - | - | - | - | - | 143 | 5.5 | 149.9 |
| 16013 | Fermented alcoholic beverage, Shaoxing wine | Beverages | Other beverages | - | - | - | - | - | 6 | 0.1 | 92.7 |
| 16014 | Distilled alcoholic beverage, "Shochu", distilled through a continuous still | Beverages | Spirits | - | - | - | - | - | 92 | 4.6 | 195.5 |
| 16015 | Distilled alcoholic beverage, "Shochu", distilled through a pot still | Beverages | Spirits | - | - | - | - | - | 258 | 10.8 | 162.5 |
| 16016 | Distilled alcoholic beverage, whisky | Beverages | Spirits | - | - | - | - | - | 33 | 0.6 | 74.6 |
| 16017 | Distilled alcoholic beverage, brandy | Beverages | Spirits | - | - | - | - | - | 6 | 0.0 | 29.5 |
| 16019 | Distilled alcoholic beverage, gin | Beverages | Spirits | - | - | - | - | - | 1 | 0.0 | 30.0 |
| 16020 | Distilled alcoholic beverage, rum | Beverages | Spirits | - | - | - | - | - | 2 | 0.0 | 2.0 |
| 16024 | Compound alcoholic beverage, "Shiro-zake" (white Sake-like liquor made from steamed rice, rice koji and "Shochu") | Beverages | Other beverages | - | - | - | - | - | 2 | 0.0 | 91.5 |
| 16025 | Compound alcoholic beverage, "Mirin" (sweet liquor made from rice, rice koji and Shochu or distilled alcohol), regular | Beverages | Mirin | - | - | - | - | - | 1663 | 2.0 | 4.8 |
| 16045 | Coffee, infusion | Beverages | Coffee | - | - | - | - | - | 2421 | 107.9 | 172.6 |
| 16046 | Coffee, instant coffee, granules | Beverages | Coffee | - | - | - | - | - | 1935 | 1.2 | 2.3 |
| 16048 | Cocoa, pure powder | Beverages | Cocoa | - | - | - | - | - | 41 | 0.0 | 3.5 |
| 16050 | "Ama-zake" (sweet beverage made from rice koji) | Beverages | Other beverages | - | - | - | - | - | 8 | 0.3 | 143.9 |
| 17004 | Hot seasoning, Doubanjiang | Seasonings and spices | Other seasonings | - | - | - | - | - | 94 | 0.1 | 2.8 |
| 17005 | Hot seasoning, hot pepper sauce | Seasonings and spices | Other seasonings | - | - | - | - | - | 18 | 0.0 | 9.2 |
| 17007 | Soy sauce, "Koikuchi-shoyu" (common soy sauce) | Seasonings and spices | Soy sauce | - | - | - | - | - | 9326 | 13.5 | 5.6 |
| 17008 | Soy sauce, "Usukuchi-shoyu" (light color soy sauce) | Seasonings and spices | Soy sauce | - | - | - | - | - | 1504 | 2.1 | 5.4 |
| 17009 | Soy sauce, "Tamari-shoyu" (full-bodied soy sauce) | Seasonings and spices | Soy sauce | - | - | - | - | - | 95 | 0.2 | 9.8 |
| 17010 | Soy sauce, "Saishikomi-shoyu" (refermented soy sauce) | Seasonings and spices | Soy sauce | - | - | - | - | - | 234 | 0.2 | 3.9 |
| 17011 | Soy sauce, "Shiro-shoyu" (extra light color soy sauce) | Seasonings and spices | Soy sauce | - | - | - | - | - | 70 | 0.1 | 7.0 |
| 17015 | Vinegar, grain vinegar | Seasonings and spices | Vinegar | - | - | - | - | - | 2023 | 3.3 | 6.3 |
| 17016 | Vinegar, rice vinegar | Seasonings and spices | Vinegar | - | - | - | - | - | 341 | 0.8 | 9.2 |
| 17017 | Vinegar, fruit vinegar, wine vinegar | Seasonings and spices | Vinegar | - | - | - | - | - | 35 | 0.2 | 17.4 |
| 17018 | Vinegar, fruit vinegar, cider vinegar | Seasonings and spices | Vinegar | - | - | - | - | - | 114 | 0.9 | 30.2 |
| 17044 | Miso, rice-koji miso, sweet type | Seasonings and spices | Miso | - | - | - | - | - | 249 | 0.7 | 11.2 |
| 17045 | Miso, rice-koji miso, light yellow type | Seasonings and spices | Miso | - | - | - | - | - | 2950 | 7.8 | 10.2 |
| 17046 | Miso, rice-koji miso, red type | Seasonings and spices | Miso | - | - | - | - | - | 532 | 1.5 | 10.9 |
| 17047 | Miso, barley-koji miso | Seasonings and spices | Miso | - | - | - | - | - | 156 | 0.3 | 8.3 |
| 17048 | Miso, soybean-koji miso | Seasonings and spices | Miso | - | - | - | - | - | 93 | 0.3 | 10.5 |
| 17053 | "Sakekasu" (sake lees) | Seasonings and spices | Sakekasu | - | - | - | - | - | 79 | 0.2 | 11.6 |
| 17083 | Yeast, baker's yeast, dried | Seasonings and spices | Yeast | - | - | - | - | - | 48 | 0.0 | 1.4 |
| Partially fermented foods | |  |  |  |  |  |  |  |  |  |  |
| 1033 | Common wheat, bread, raisin bread | Cereals | Breads | 1028 | Bread, white long roll | - | 0.82 | 1 | 50 | 0.8 | 58.4 |
| 1035 | Common wheat, bread, croissants | Cereals | Breads | 1028 | Bread, white long roll | - | 0.86 | 1 | 56 | 0.9 | 60.3 |
| 1112 | Rice, non-glutinous rice products, "Yaki-onigiri" (baked rice ball) | Cereals | Soy sauce | 17007 | Soy sauce, "Koikuchi-shoyu" | - | 0.07 | 1 | 4 | 0.1 | 72.5 |
| 4031 | Soybeans, "Budo-mame" (beans cooked with sugar and salt) | Pulses | Mirin Soy sauce | 16025 17007 | "Mirin" Soy sauce, "Koikuchi-shoyu" | 0.06 0.18 | 0.24 | 3b | 41 | 0.3 | 28.6 |
| 6067 | Cucumber, fruit, pickles, pickled in soy sauce | Vegetables | Soy sauce | 17007 | Soy sauce, "Koikuchi-shoyu" | - | 0.28 | 2 | 55 | 0.3 | 19.3 |
| 6069 | Cucumber, fruit, pickles, sweet type (pickled with seasoned vinegar) | Vegetables | Vinegar | 17015 | Vinegar, grain vinegar | - | 0.32 | 3a | 56 | 0.1 | 5.4 |
| 6104 | Ginger, mature rhizome, pickles | Vegetables | Vinegar | 17015 | Vinegar, grain vinegar | - | 0.14 | 2 | 187 | 0.3 | 5.5 |
| 6105 | Ginger, mature rhizome, pickles, sweetened | Vegetables | Vinegar | 17015 | Vinegar, grain vinegar | - | 0.19 | 2 | 148 | 0.3 | 8.9 |
| 6141 | Japanese radishes, Daikon, root, pickles, "Bettara-zuke" (pickled with rice koji) | Vegetables | Rice-koji | 1116 | Rice-koji | - | 0.13 | 3b | 28 | 0.2 | 28.4 |
| 6142 | Japanese radishes, Daikon, root, pickles, "Miso-zuke" (pickled with miso) | Vegetables | Miso | 17045 | Miso, rice-koji miso, light yellow type | - | 0.90 | 2 | 14 | 0.0 | 13.1 |
| 6143 | Japanese radishes, Daikon, root, pickles, "Fukujin-zuke" (pickled with Daikon, eggplant, immature sword pods and east Indian lotus rhizome ) | Vegetables | Soy sauce Vinegar | 17007 17015 | Soy sauce, "Koikuchi-shoyu" Vinegar, grain vinegar | 0.35 0.12 | 0.47 | 2 | 111 | 0.4 | 14.6 |
| 6197 | Eggplant*, pickles, "Koji-zuke" (pickled small oval type with rice koji ) [*Syn. Aubergine] | Vegetables | Rice-koji Soy sauce | 1116 17007 | Rice-koji Soy sauce, "Koikuchi-shoyu" | 0.09 0.10 | 0.19 | 3b | 2 | 0.0 | 21.0 |
| 6198 | Eggplant*, pickles, "Karashi-zuke" (pickled small oval type with mustard) [*Syn. Aubergine] | Vegetables | Sakekasu | 17053 | "Sakekasu" | - | 0.11 | 3b | 5 | 0.1 | 40.0 |
| 6231 | Turnip green, "Nozawana", leaves, pickles, seasoned | Vegetables | Soy sauce Vinegar | 17007 17015 | Soy sauce, "Koikuchi-shoyu" Vinegar, grain vinegar | 0.16 0.13 | 0.19 | 2 | 85 | 0.7 | 29.9 |
| 6253 | Turnip, "Hinona", root with tops, pickles, sweetened | Vegetables | Vinegar | 17015 | Vinegar, grain vinegar | - | 0.14 | 4 | 3 | 0.0 | 11.0 |
| 6295 | Thistle root, "Yamagobo", pickled with miso | Vegetables | Miso Mirin | 17045 16025 | Miso, rice-koji miso, light yellow type "Mirin" | 0.37 0.06 | 0.43 | 3b | 4 | 0.0 | 23.0 |
| 6306 | Japanese scallion, "Rakkyo", mature bulb, pickles, sweetened | Vegetables | Vinegar | 17015 | Vinegar, grain vinegar | - | 0.10 | 2 | 176 | 0.9 | 19.4 |
| 6323 | Wasabi, pickled with Sake lees | Vegetables | Sakekasu | 17053 | "Sakekasu" | - | 0.50 | 1 | 29 | 0.1 | 9.2 |
| 7039 | Olives, in brine, stuffed olives | Fruits | Pickles | 7037 | Olives, in brine, green | - | 0.75 | 1 | 2 | 0.0 | 8.0 |
| 8003 | Mushrooms, winter mushrooms*, bottled in seasoning [*Syn. Enokitake, Enoki] | Mushrooms | Soy sauce | 17007 | Soy sauce, "Koikuchi-shoyu" | - | 0.10 | 2 | 15 | 0.1 | 16.1 |
| 9005 | Algae, purple laver, dried, seasoned and toasted | Algae | Soy sauce | 17007 | Soy sauce, "Koikuchi-shoyu" | - | 0.21 | 2 | 450 | 0.2 | 1.6 |
| 9022 | Algae, kombu, "Shio-kombu" (seasoned and dried) | Algae | Soy sauce | 17007 | Soy sauce, "Koikuchi-shoyu" | - | 0.20 | 2 | 55 | 0.1 | 4.4 |
| 9023 | Algae, kombu, "Tsukudani" (simmered in soy sauce and sugar) | Algae | Soy sauce | 17007 | Soy sauce, "Koikuchi-shoyu" | - | 0.28 | 2 | 190 | 0.5 | 9.2 |
| 9033 | Algae, "Hitoegusa", "Tsukudani" (simmered in soy sauce and sugar) | Algae | Soy sauce | 17007 | Soy sauce, "Koikuchi-shoyu" | - | 0.18 | 2 | 73 | 0.2 | 8.1 |
| 10035 | Fish, Japanese sand lance, "Tsukudani" (simmered whole in soy sauce and sugar) | Fish, mollusks and crustaceans | Soy sauce | 17007 | Soy sauce, "Koikuchi-shoyu" | - | 0.29 | 2 | 27 | 0.1 | 12.8 |
| 10036 | Fish, Japanese sand lance, "Ameni" (simmered whole in glucose syrup and soy sauce) | Fish, mollusks and crustaceans | Soy sauce | 17007 | Soy sauce, "Koikuchi-shoyu" | - | 0.30 | 2 | 6 | 0.0 | 8.2 |
| 10058 | Fish, sardine, Japanese anchovy, "Mirinboshi" (split seasoned with Mirin and dried) | Fish, mollusks and crustaceans | Mirin Soy sauce | 16025 17007 | "Mirin" Soy sauce, "Koikuchi-shoyu" | 0.07 0.07 | 0.14 | 2 | 7 | 0.0 | 18.0 |
| 10059 | Fish, sardine, Japanese pilchard, "Mirinboshi" (split seasoned with Mirin and dried) | Fish, mollusks and crustaceans | Mirin Soy sauce | 16025 17007 | "Mirin" Soy sauce, "Koikuchi-shoyu" | 0.12 0.12 | 0.24 | 2 | 9 | 0.1 | 22.8 |
| 10061 | Fish, sardine, Japanese pilchard, canned products, with seasoning | Fish, mollusks and crustaceans | Soy sauce | 17007 | Soy sauce, "Koikuchi-shoyu" | - | 0.04 | 2 | 16 | 0.2 | 43.4 |
| 10064 | Fish, sardine, Japanese pilchard, "Kabayaki" (baked and seasoned fillet), canned products, | Fish, mollusks and crustaceans | Soy sauce | 17007 | Soy sauce, "Koikuchi-shoyu" | - | 0.05 | 2 | 1 | 0.0 | 35.0 |
| 10070 | Fish, eel, "Kabayaki" (seasoned and baked fillet) | Fish, mollusks and crustaceans | Soy sauce | 17007 | Soy sauce, "Koikuchi-shoyu" | - | 0.07 | 2 | 112 | 1.7 | 58.5 |
| 10072 | Fish, black scraper*, "Ajitsuke-hirakiboshi" (seasoned and dried fillet) [*Syn. filefish] | Fish, mollusks and crustaceans | Soy sauce | 17007 | Soy sauce, "Koikuchi-shoyu" | - | 0.29 | 2 | 2 | 0.0 | 25.0 |
| 10093 | Fish, skipjack tuna and frigate mackerel, processed products, "Kezuri-bushi" (shaved "Katsuo-bushi"), simmered in soy sauce and sugar (cf. 10091"Katsuo-bushi") | Fish, mollusks and crustaceans | Soy sauce | 10092 17007 | "Kezuri-bushi" Soy sauce, "Koikuchi-shoyu" | 0.49 0.50 | 0.99 | 2 | 20 | 0.0 | 5.8 |
| 10094 | Fish, skipjack tuna and frigate mackerel, processed products, "Kakuni" (meat cube boiled in soy sauce and sugar) | Fish, mollusks and crustaceans | Soy sauce | 17007 | Soy sauce, "Koikuchi-shoyu" | - | 0.24 | 2 | 3 | 0.0 | 19.7 |
| 10125 | Fish, dotted gizzard shad*, "Amazu-zuke" (marinated in vinegar and sugar) [*Syn. gizzard shad] | Fish, mollusks and crustaceans | Vinegar | 17015 | Vinegar, grain vinegar | - | 0.14 | 4 | 3 | 0.0 | 22.3 |
| 10163 | Fish, mackerels, processed products, "Shimesaba" (vinegar marinated fillet) | Fish, mollusks and crustaceans | Vinegar | 17015 | Vinegar, grain vinegar | - | 0.14 | 4 | 23 | 0.2 | 29.0 |
| 10165 | Fish, mackerel, canned products, boiled with miso | Fish, mollusks and crustaceans | Miso | 17045 | Miso, rice-koji miso, light yellow type | - | 0.07 | 2 | 8 | 0.2 | 78.1 |
| 10166 | Fish, mackerel, canned products, boiled with seasoning | Fish, mollusks and crustaceans | Soy sauce | 17007 | Soy sauce, "Koikuchi-shoyu" | - | 0.07 | 2 | 1 | 0.0 | 180.0 |
| 10176 | Fish, Pacific saury, "Mirinboshi" (seasoned with Mirin and dried fillet) | Fish, mollusks and crustaceans | Mirin Soy sauce | 16025 17007 | "Mirin" Soy sauce, "Koikuchi-shoyu" | 0.18 0.18 | 0.36 | 2 | 3 | 0.0 | 47.7 |
| 10177 | Fish, Pacific saury, canned products, boiled with seasoning | Fish, mollusks and crustaceans | Soy sauce | 17007 | Soy sauce, "Koikuchi-shoyu" | - | 0.08 | 2 | 2 | 0.0 | 70.0 |
| 10178 | Fish, Pacific saury, canned products, "Kabayaki" (baked and seasoned fillet) | Fish, mollusks and crustaceans | Soy sauce | 17007 | Soy sauce, "Koikuchi-shoyu" | - | 0.08 | 2 | 6 | 0.1 | 44.7 |
| 10210 | Fish, cod, Pacific cod, "Denbu" (mashed and seasoned meat) | Fish, mollusks and crustaceans | Sake Mirin Soy sauce | 16001 16025 17007 | "Sake", regular "Mirin" Soy sauce, "Koikuchi-shoyu" | 0.07 0.03 0.05 | 0.15 | 3b | 43 | 0.0 | 2.6 |
| 10240 | Fish, crucian carp, "Kanroni" (simmered whole in soy sauce, sugar and glucose syrup) | Fish, mollusks and crustaceans | Sake Soy sauce | 16001 17007 | "Sake", regular Soy sauce, "Koikuchi-shoyu" | 0.12 0.12 | 0.24 | 3b | 1 | 0.0 | 60.0 |
| 10277 | Fish, Japanese smelt*, "Tsukudani" (simmered whole in soy sauce and sugar) [*Syn. Pond smelt] | Fish, mollusks and crustaceans | Sake Mirin Soy sauce | 16001 16025 17007 | "Sake", regular "Mirin" Soy sauce, "Koikuchi-shoyu" | 0.10 0.04 0.09 | 0.40 | 3b | 1 | 0.0 | 15.0 |
| 10278 | Fish, Japanese smelt*, "Ameni" (simmered whole in glucose syrup and soy sauce) [*Syn. Pond smelt] | Fish, mollusks and crustaceans | Sake Mirin Soy sauce | 16001 16025 17007 | "Sake", regular "Mirin" Soy sauce, "Koikuchi-shoyu" | 0.10 0.04 0.09 | 0.23 | 3b | 1 | 0.0 | 15.0 |
| 10282 | Mollusks, short-necked clam*, "Tsukudani" (simmered meat in soy sauce and sugar) [*Syn. baby-neck clam, Manila clam, Japanese littleneck] | Fish, mollusks and crustaceans | Sake Mirin Soy sauce | 16001 16025 17007 | "Sake", regular "Mirin" Soy sauce, "Koikuchi-shoyu" | 0.12 0.09 0.09 | 0.30 | 3b | 14 | 0.0 | 11.1 |
| 10284 | Mollusks, short-necked clam*, canned products, boiled with seasoning  [*Syn. baby-neck clam, Manila clam, Japanese littleneck] | Fish, mollusks and crustaceans | Soy sauce | 17007 | Soy sauce, "Koikuchi-shoyu" | - | 0.04 | 2 | 3 | 0.0 | 50.0 |
| 10318 | Mollusks, ark shell*, canned with seasoning [*Syn. Mogai clam] | Fish, mollusks and crustaceans | Soy sauce | 17007 | Soy sauce, "Koikuchi-shoyu" | - | 0.08 | 2 | 3 | 0.0 | 25.3 |
| 10331 | Crustacean, processed shrimp, "Tsukudani" (simmered whole in soy sauce and sugar) | Fish, mollusks and crustaceans | Sake Mirin Soy sauce | 16001 16025 17007 | "Sake", regular "Mirin" Soy sauce, "Koikuchi-shoyu" | 0.09 0.17 0.16 | 0.42 | 3b | 9 | 0.0 | 3.4 |
| 10351 | Mollusks, firefly squid, "Tsukudani" (simmered whole in soy sauce and sugar) | Fish, mollusks and crustaceans | Sake Mirin Soy sauce | 16001 16025 17007 | "Sake", regular "Mirin" Soy sauce, "Koikuchi-shoyu" | 0.05 0.12 0.15 | 0.32 | 3b | 1 | 0.0 | 10.0 |
| 10359 | Mollusks, processed squid, canned with seasoning | Fish, mollusks and crustaceans | Soy sauce | 17007 | Soy sauce, "Koikuchi-shoyu" | - | 0.10 | 2 | 1 | 0.0 | 50.0 |
| 10363 | Crustacean, opossum shrimp*, "Tsukudani" (simmered whole in soy sauce and sugar)[*Syn. Mysids] | Fish, mollusks and crustaceans | Soy sauce | 17007 | Soy sauce, "Koikuchi-shoyu" | - | 0.43 | 3b | 5 | 0.0 | 19.0 |
| 11106 | Beef products, canned with seasoning | Meat | Sake Mirin Soy sauce | 16001 16025 17007 | "Sake", regular "Mirin" Soy sauce, "Koikuchi-shoyu" | 0.11 0.08 0.11 | 0.30 | 3b | 1 | 0.0 | 50.0 |
| 11195 | Pork, roast pork | Meat | Soy sauce | 17007 | Soy sauce, "Koikuchi-shoyu" | - | 0.16 | 2 | 152 | 1.1 | 29.0 |
| 11237 | Chicken, canned products, roast meat with seasoning | Meat | Soy sauce | 17007 | Soy sauce, "Koikuchi-shoyu" | - | 0.07 | 2 | 1 | 0.0 | 16.0 |
| 12017 | Eggs, hen, Tamago-dofu (cold savory egg custard) | Eggs | Mirin Soy sauce | 16025 17007 | "Mirin" Soy sauce, "Koikuchi-shoyu" | 0.04 0.01 | 0.05 | 1 | 24 | 0.5 | 84.1 |
| 12018 | Eggs, hen, Tamago-yaki (Rolled omelet), Atsuyaki-tamago (sweet rolled omelet with Katsuo-bushi and kombu dashi) | Eggs | Soy sauce | 17008 | Soy sauce, "Usukuchi-shoyu" | - | 0.01 | 1 | 59 | 0.5 | 32.9 |
| 12019 | Eggs, hen, Tamago-yaki (Rolled omelet), Dashimaki-tamago (rolled omelet with Katsuo-bushi and kombu dashi) | Eggs | Soy sauce | 17008 | Soy sauce, "Usukuchi-shoyu" | - | 0.02 | 1 | 31 | 0.2 | 27.6 |
| 13041 | Cheeses, spreadable | Milk and milk products | Cheese | 13040 | Cheeses, processed | - | 0.30 | 3c | 1 | 0.0 | 10.0 |
| 15019 | Traditional confectionery, "Kushi-dango, soy sauce" (skewered rice dumplings, steamed, seasoned with sugar and soy sauce) | Confectionaries | Soy sauce | 17007 | Soy sauce, "Koikuchi-shoyu" | - | 0.06 | 1 | 21 | 0.4 | 73.8 |
| 15032 | Traditional confectionery, "To-manju" (baked sweet dough stuffed with sweet adzuki bean paste) | Confectionaries | Mirin | 16025 | "Mirin" | - | 0.01 | 1 | 37 | 0.4 | 39.3 |
| 15034 | Chinese style steamed bun, stuffed with sweet adzuki bean paste | Confectionaries | Breads | 1028 | Bread, white long roll | - | 0.59 | 1 | 4 | 0.1 | 86.5 |
| 15035 | Chinese style steamed bun, stuffed with meat and vegetable | Confectionaries | Breads | 1028 | Bread, white long roll | - | 0.69 | 1 | 42 | 1.1 | 97.3 |
| 15058 | Traditional confectionery, "Amakara-senbei" (rice cracker coated with soy sauce and grain sugar) | Confectionaries | Soy sauce | 17007 | Soy sauce, "Koikuchi-shoyu" | - | 0.08 | 1 | 53 | 0.3 | 22.0 |
| 15059 | Traditional confectionery, "Arare" (glutinous rice cracker) | Confectionaries | Soy sauce | 17007 | Soy sauce, "Koikuchi-shoyu" | - | 0.11 | 1 | 134 | 0.6 | 18.1 |
| 15060 | Traditional confectionery, "Shoyu-senbei" (soy sauce flavored rice cracker) | Confectionaries | Soy sauce | 17007 | Soy sauce, "Koikuchi-shoyu" | - | 0.09 | 1 | 141 | 0.7 | 19.6 |
| 15069 | Bun with filling, baked bun with sweet adzuki bean paste filling regular type | Confectionaries | Breads | 1028 | Bread, white long roll | - | 0.59 | 1 | 138 | 2.4 | 68.0 |
| 15070 | Bun with filling, baked bun with custard cream filling regular type | Confectionaries | Breads | 1028 | Bread, white long roll | - | 0.63 | 1 | 84 | 1.2 | 54.3 |
| 15071 | Bun with filling, baked bun with strawberry jam filling | Confectionaries | Breads | 1028 | Bread, white long roll | - | 0.63 | 1 | 38 | 0.3 | 32.8 |
| 15072 | Bun with filling, "Korone" (horn-shaped bread), with chocolate cream filling | Confectionaries | Breads | 1028 | Bread, white long roll | - | 0.56 | 1 | 69 | 0.8 | 46.5 |
| 15076 | Cake and pastry, Danish pastry | Confectionaries | Breads | 1028 | Bread, white long roll | - | 0.69 | 1 | 61 | 1.1 | 68.2 |
| 15088 | Pudding and chilled dessert, coffee jelly | Confectionaries | Coffee | 16046 | Coffee, instant coffee, granules | - | 0.01 | 1 | 22 | 0.5 | 91.5 |
| 15090 | Pudding and chilled dessert, wine flavored jelly | Confectionaries | Wine | 16011 | Wine, red | - | 0.1 | 1 | 1 | 0.0 | 20.0 |
| 15114 | Chocolate, chocolate-covered biscuit | Confectionaries | Cocoa | 16048 | Cocoa, pure powder | - | 0.21 | 1 | 64 | 0.4 | 23.6 |
| 15116 | Chocolate, milk chocolate | Confectionaries | Cocoa | 16048 | Cocoa, pure powder | - | 0.35 | 3c | 324 | 1.5 | 18.3 |
| 16022 | Compound alcoholic beverage, "Umeshu" (plum liquor made from Japanese apricots) | Beverages | Sake | 16001 | "Sake", regular | - | 0.51 | 1 | 25 | 0.5 | 70.6 |
| 16023 | Compound alcoholic beverage, synthetic "Sake" | Beverages | Sake | 16001 | "Sake", regular | - | 0.07 | 3c | 141 | 0.2 | 5.8 |
| 16026 | Compound alcoholic beverage, "Honnaoshi" (sweet liquor made of "Shochu" and "Mirin") | Beverages | Mirin | 16025 | "Mirin" | - | 0.55 | 2 | 4 | 0.0 | 23.0 |
| 16027 | Compound alcoholic beverage, medicinal liqueur | Beverages | Mirin | 16025 | "Mirin" | - | 0.9 | 5 | 6 | 0.0 | 11.2 |
| 16028 | Compound alcoholic beverage, curacao | Beverages | Spirits | 16017 | Distilled alcoholic beverage, brandy | - | 0.9 | 5 | 1 | 0.0 | 3.3 |
| 16029 | Compound alcoholic beverage, fortified wine, sweet type | Beverages | Wine | 16011 | Wine, red | - | 0.12 | 2 | 7 | 0.2 | 101.6 |
| 16047 | Coffee, ready-to-drink coffee with milk and sugar, canned | Beverages | Coffee | 16045 | Coffee, infusion | - | 0.28 | 2 | 370 | 18.3 | 191.0 |
| 16049 | Cocoa, chocolate milk powder | Beverages | Cocoa | 16048 | Cocoa, pure powder | - | 0.41 | 2 | 99 | 0.3 | 12.1 |
| 17001 | Japanese Worcester sauce, common type | Seasonings and spices | Vinegar | 17015 | Vinegar, grain vinegar | - | 0.30 | 3c | 372 | 0.9 | 9.4 |
| 17002 | Japanese Worcester sauce, semi-thick type | Seasonings and spices | Vinegar | 17015 | Vinegar, grain vinegar | - | 0.30 | 3c | 62 | 0.2 | 12.7 |
| 17003 | Japanese Worcester sauce, thick type | Seasonings and spices | Vinegar | 17015 | Vinegar, grain vinegar | - | 0.30 | 3c | 277 | 1.0 | 13.9 |
| 17019 | Soup stock, "Katsuo-bushi dashi" (stock of "Katsuo-bushi") (cf. 10091"Katsuo-bushi") | Seasonings and spices | Katsuo-bushi | 10092 | "Kezuri-bushi" | - | 0.007 | 2 | 1504 | 45.4 | 116.9 |
| 17021 | Soup stock, "Katsuo-bushi and kombu dashi" (stock of "Katsuo-bushi" and dried kombu) (cf. 10091"Katsuo-bushi") | Seasonings and spices | Katsuo-bushi | 10092 | "Kezuri-bushi" | - | 0.003 | 2 | 846 | 18.4 | 84.2 |
| 17028 | Stock powder, "Katsuo-bushi" (cf. 10091"Katsuo-bushi") | Seasonings and spices | Katsuo-bushi | 10092 | "Kezuri-bushi" | - | 0.31 | 2 | 2577 | 0.9 | 1.3 |
| 17029 | Japanese noodle soup, non-concentrated (soy sauce base) | Seasonings and spices | Mirin Soy sauce | 16025 17007 | "Mirin" Soy sauce, "Koikuchi-shoyu" | 0.17 0.16 | 0.33 | 3b | 910 | 11.5 | 48.8 |
| 17030 | Japanese noodle soup, triple-concentrated (soy sauce base) | Seasonings and spices | Mirin Soy sauce | 16025 17007 | "Mirin" Soy sauce, "Koikuchi-shoyu" | 0.50 0.49 | 0.99 | 3b | 313 | 1.1 | 13.1 |
| 17032 | Seasoning sauce, Mapo tofu sauce | Seasonings and spices | Other seasonings | 17004 | Hot seasoning, Doubanjiang | - | 0.19 | 3b | 35 | 0.4 | 48.2 |
| 17036 | Tomato products, ketchup | Seasonings and spices | Vinegar | 17015 | Vinegar, grain vinegar | - | 0.17 | 2 | 525 | 1.4 | 10.4 |
| 17038 | Tomato products, chili sauce | Seasonings and spices | Sake Vinegar Other seasonings | 16001 17004 17015 | "Sake", regular Vinegar, grain vinegar Hot seasoning, Doubanjiang | 0.08 0.14 0.04 | 0.38 | 3b | 33 | 0.2 | 20.3 |
| 17039 | Dressing, soy sauce based, fat-free | Seasonings and spices | Soy sauce Vinegar Mirin | 16025 17007 17015 | "Mirin" Soy sauce, "Koikuchi-shoyu" Vinegar, grain vinegar | 0.06 0.24 0.19 | 0.49 | 1 | 553 | 1.5 | 10.7 |
| 17040 | Dressing, French dressing | Seasonings and spices | Vinegar | 17015 | Vinegar, grain vinegar | - | 0.36 | 1 | 360 | 1.0 | 10.6 |
| 17041 | Dressing, thousand island dressing | Seasonings and spices | Vinegar | 17015 | Vinegar, grain vinegar | - | 0.12 | 2 | 107 | 0.3 | 11.6 |
| 17042 | Dressing, mayonnaise, whole egg type | Seasonings and spices | Vinegar | 17015 | Vinegar, grain vinegar | - | 0.12 | 2 | 356 | 0.8 | 8.6 |
| 17043 | Dressing, mayonnaise, egg yolk type | Seasonings and spices | Vinegar | 17015 | Vinegar, grain vinegar | - | 0.12 | 2 | 1091 | 2.5 | 8.8 |
| 17049 | Miso, instant miso soup, powdered type | Seasonings and spices | Miso Katsuo-bushi | 17045 10092 | Miso, rice-koji miso, light yellow type "Kezuri-bushi" | 0.97 0.01 | 0.98 | 5 | 2 | 0.0 | 5.0 |
| 17050 | Miso, instant miso soup, Paste type | Seasonings and spices | Miso Katsuo-bushi | 17045 10092 | Miso, rice-koji miso, light yellow type "Kezuri-bushi" | 0.97 0.01 | 0.98 | 5 | 55 | 0.2 | 13.5 |
| 17059 | Spices, mustard, yellow mustard | Seasonings and spices | Vinegar | 17015 | Vinegar, grain vinegar | - | 0.48 | 2 | 47 | 0.0 | 2.1 |
| 17060 | Spices, mustard, whole grain mustard | Seasonings and spices | Vinegar | 17015 | Vinegar, grain vinegar | - | 0.29 | 2 | 14 | 0.0 | 7.0 |
| 18013 | Hamburger steak, frozen | Prepared food | Breads | 1077 | Bread crumbs, fresh | - | 0.06 | 3a | 8 | 0.1 | 52.5 |

STFCJ, Standard Tables of Food Composition in Japan

* Mean intake (g/day) = total intake of each food / total number of days of dietary records (3,872days)

† Mean intake (g/time) = total intake of each food / total number of appearances
